# Volume Change during Creep and Micromechanical Deformation Processes in PLA–PBSA Binary Blends

**DOI:** 10.3390/polym13142379

**Published:** 2021-07-20

**Authors:** Laura Aliotta, Vito Gigante, Maria-Beatrice Coltelli, Andrea Lazzeri

**Affiliations:** 1 Department of Civil and Industrial Engineering, University of Pisa, 56122 Pisa, Italy; vito.gigante@dici.unipi.it (V.G.); maria.beatrice.coltelli@unipi.it (M.-B.C.); 2 National Interuniversity Consortium of Materials Science and Technology (INSTM), 50121 Florence, Italy

**Keywords:** binary blends, creep, micromechanics

## Abstract

In this paper, creep measurements were carried out on poly(lactic acid) (PLA) and its blends with poly(butylene succinate-adipate) (PBSA) to investigate the specific micromechanical behavior of these materials, which are promising for replacing fossil-based plastics in several applications. Two different PBSA contents at 15 and 20 wt.% were investigated, and the binary blends were named 85-15 and 80-20, respectively. Measurements of the volume strain, using an optical extensometer, were carried out with a universal testing machine in creep configuration to determine, accompanied by SEM images, the deformation processes occurring in a biopolymeric blend. With the aim of correlating the creep and the dilatation variation, analytical models were applied for the first time in biopolymeric binary blends. By using an Eyring plot, a significant change in the curves was found, and it coincided with the onset of the cavitation/debonding mechanism. Furthermore, starting from the data of the pure PLA matrix, using the Eyring relationship, an apparent stress concentration factor was calculated for PLA-PBSA systems. From this study, it emerged that the introduction of PBSA particles causes an increment in the apparent stress intensity factor, and this can be ascribed to the lower adhesion between the two biopolymers. Furthermore, as also confirmed by SEM analysis, it was found that debonding was the main micromechanical mechanism responsible for the volume variation under creep configuration; it was found that debonding starts earlier (at a lower stress level) for the 85-15 blend.

## 1. Introduction

Physical polymeric blends, constituted by a rubbery phase embedded in a more rigid matrix, are a long-lasting route for the preparation of new materials with a modulated balance of properties [1]. The advent of biobased and biodegradable polymers could positively affect the environmental profile of products, thanks to an improved carbon neutrality with respect to fossil-based counterparts and a more environmentally friendly end of life [2,3,4]. The increased interest towards biopolymers has resulted in a revival of blending technology and, in order to exploit their potential and enter new markets, the study of these materials is at the center of scientific research [5]. Improved knowledge of the mechanical behavior of biobased materials is fundamental for better exploiting their peculiar properties and comparing them with fossil-based ones, favoring the replacement of the latter in several application sectors.

In this context, it is beneficial to investigate micromechanical deformation processes that occur in physical polymeric blends, knowing that external stresses can be the cause of the starting point of numerous micromechanical deformation processes that play a critical role regarding the failure of pure heterogeneous systems [6]. The two basic micromechanical deformation processes in pure polymers are shear yielding and crazing [7], while in multiphase systems, it can also be active debonding and cavitation [8]. The debonding mechanism involves the formation of cavities/voids at the interface between the rubber phase and the matrix, while void formation occurring internally to the rubber particles is known as cavitation [9]. The expansion of the cavities occurs when the volumetric strain energy is greater than that required for the creation of the void surface area [10]. The parameter governing the cavitation or debonding mechanism is the value of the polymer/rubber interfacial adhesion: high adhesion values contribute to the internal cavitation of the rubber particles, while low values contribute to the debonding mechanism [11]. It is important to state that micromechanical deformations are competitive processes, the prevailing one is determined by the inherent properties of the matrix polymer and by local stress distribution [12]. The role and importance of void formation within or around the rubber particles in polymer blends are still not clear, but they have been at the center of academic interest in material science [13,14,15,16]. On the basis of energy balances, Lazzeri and Bucknall [17,18] developed the idea that cavitation/debonding of rubber particles arises at the crack tip and can be the cause of dilatational shear yielding and/or crazing in the matrix. The following matrix distortion is not homogeneous and becomes highly localized due to the formation of bands of voids and sheared material called “dilatational shear bands” or more simply “dilatational bands”. The effect of cavitation is a local decreasing of the bulk modulus and hydrostatic stress components near the void and a corresponding growth of the stress deviatoric component. Higher elastic energy may then cause a faster advance of shear bands and, thus, a larger plastic zone form is attained [19].

In order to understand the micromechanical deformation processes that occur in particular polymeric blends, measurements and analysis of the volume change during uniaxial tensile or creep tests can lead to a better understanding of the deformation phenomena [20,21]. In particular, the theories existing in literature state that during shear yielding, the volume of the sample remains constant, while crazing, debonding, and cavitation are characterized by the increase in volume strain [22]. To make a “quantitative evaluation” of the deformation mechanisms effective in rubber-toughened systems, dilatometric studies of tensile creep have been carried out as function of the tensile stress or strain prior to fracture [23].

During uniaxial tensile testing, after that the rubber particles generate voids (by cavitation and or debonding), the voids elongate as the specimen extends. Generally, debonding or cavitation occurs before yielding [24]. The void growth mechanism is a second and successive deformation process that causes great differences in volume variation. Since this process occurs after yielding and it is in common to both cavitation and debonding phenomena, very interesting are creep studies in which different stress levels (below the yield stress) are investigated in order to identify the stress level for which the debonding or cavitation process begins.

In tensile creep experiments, the onset of dilatational yielding goes with a rapid increase in deformation; for this reason, the void volume will increase with strain in addition to the volume change of the matrix itself [20]. To avoid the drawbacks of mechanical extensometers, which include range limitations, recently video-controlled tensile testing equipment has been developed by G’Sell et al. [25,26] to optically evaluate the volumetric strain.

The abovementioned important concepts have been tested and developed on conventional polymeric systems; however, to the best of our knowledge, they have not been extensively explored on biopolymeric blends. In particular, coupling between the dilatometric volume measurement with an optical system performed in real time during creep tests of a biopolymeric blend is a novelty.

The present paper, starting from one of the author’s own experience [27,28], aimed to explore the micromechanical deformation mechanisms of blends based on polylactic acid (PLA) and polybutylene succinate adipate (PBSA). These blends were recently considered for their potential use in packaging and personal care/sanitary applications [29], hence, in sectors where products have a short life thus highly contributing to the production of enormous amounts of waste [30]. In this paper, two formulations were studied in which PBSA was introduced at 15 and 20 wt.%, respectively.

Krishnan et al. [31], LeBarbe [32], and Nagarjan et al. [33] published exhaustive reviews regarding the problem of PLA toughening and the necessity of increasing the ductility without losing too many of the characteristics that make PLA interesting, i.e., high elastic modulus, good processability, high tensile strength (without forgetting biodegradability and biobased content).

In any case, while different researchers have investigated PLA toughened with biodegradable rubber, in which they stated that certainly the size of the dispersed particles, together with the quality of interfacial adhesion, determines the final toughening effect in PLA [34,35,36,37,38,39,40], only a few papers studied the short-time creep behavior of PLA-based blends [41,42,43], and none of them addressed the issue of relating micromechanical deformation phenomena with volumetric dilatometric variations from creep tests. For this reason, in this work, measurements of the volume strain, using an optical extensometer, were conducted with a universal testing machine in creep configuration to determine, accompanied by SEM images, the micromechanical deformation processes involved in a biopolymeric blend system.

Analytical models were also applied in order to correlate the creep to the dilatation variation; in particular, the Andrade equation [8] was applied and the *b* parameter for the polymeric systems was calculated. Plotting the *log b* against the applied stress, using an Eyring plot, a significant change in the curves was found, and it coincided with the onset of the cavitation/debonding mechanism. Furthermore, knowing the data of the pure matrix, from the Eyring relationship, the apparent stress concentration factor was calculated for the PLA binary blends with 15 and 20 wt.% of PBSA.

## 2. Theoretical Analysis and Theoretical Background

Creep rupture of a polymer is the result of combined events (such as viscoelastic deformation, primary and secondary bond rupture, shear yielding, crazing, void formation and growth) and fibril breakdown with intrinsic and extrinsic flaws, leading to fracture. In the case of polymer blends or composites, the interfacial strength and morphology must also be taken into account [44]. According to the creep curves (*creep strain* (*ε*) *vs. creep time* (*t*)), in polymers, four stages can be considered [45]: (I) the first stage of instantaneous deformation (ε_0_), (II) the second stage named primary creep (ε_1_), (III) the third stage named secondary or transient creep (ε_2_) in which the creep rate reaches a steady-state value, and the fourth stage (IV) (ε_4_) in which the creep rate increases abruptly and the final creep rupture occurs. Transient creep of many materials (including polymeric materials) obeys Andrade’s law in which creep strain is proportional to the cube root of time according to the following equation [46,47,48]:

(1) $$\varepsilon \left(t\right)=\varepsilon \left(0\right)+bt^{\frac{1}{3}}$$

where *ε*(*0*) is the time independent instantaneous elongation due to the elastic or plastic deformation of polymer once the external load is applied; b is a function of stress and temperature. The validity of the Andrade’s approach can be easily verified by plotting in the region of the transient creep, *ε*(*t*) against *t*^1/3^, and checking if the data align on a straight line. If the data fits the model, the *b* parameter can be thus obtained, because it is equal to the angular coefficient of the straight line.

If the *b* parameter is known, it can be related to the activation volume according to the following Eyring relationship [48]:

(2) $$b=Q\exp\left(\frac{\gamma V\sigma }{k_{B}T}\right)$$

where *Q* is a constant, γ is a stress concentration factor, *V* is the activation volume for the deformation process, *k_B_* Boltzmann’s constant, and *T* the temperature. Making the assumption that in the absence of rubber particles (or other stress-concentrating additives like rigid fillers), γ = 1 and the activation volume can be easily calculated for the pure matrix. Once the activation volume is obtained, the variation in the apparent stress concentration factor induced by the addition of the rubber particles to the matrix can be obtained following the procedure explained in [48].

In [8], it was shown that Eyring plots of log *b* against applied stress were linear for the pure polyamide (PA66), but for the rubber toughened polyamide (RTPA66), it showed a sharp increase in *d* log *b*/*dσ*, where significant dilatation begins. Matching the results of creep tests and scanning electron micrographs, it was concluded that this cavitation accelerates shear yielding in the nylon matrix. The main explanation for this behavior was correlated with the energy-balance model for cavitation combined with the modified version of Gurson’s equation for dilatation at yielding. According to the energy-balance model, the critical volume deformation Δ*_v_^c^* above which a particle can cavitate can be determined by Equation (3) [18]:

(3) $$\Delta_{v}^{c}=4{\left(\frac{4\Gamma }{3K_{r}D}\right)}^{3/4}$$

where Γ is the surface energy of the rubber, *D* the particle diameter, and *K_r_* the rubber bulk modulus. When a rubber-toughened material is subjected to an external load, during the earlier stages of deformation, the hydrostatic component of the stress in the material starts to build up and, at a certain point, the biggest particles will start to cavitate and/or to debond. In this initial stage, voids will appear randomly, but their presence significantly affects the yielding and fracture behavior of polymers. If the particles cavitate, Lazzeri and Bucknall [17,18] proposed a modified version of the Gurson yield function [49] to account for the effects of cavitation on the yielding behavior of rubber-toughened polymers:

(4) $$\sigma_{e}^{2}=\sigma_{\phi }^{2}\left[\left(1-\frac{\mu \sigma_{m}}{\sigma_{\phi }}\right)-2fq_{1}\cosh\left(\frac{3q_{2}\sigma_{m}}{2\sigma_{\phi }}\right)+{\left(q_{1}f\right)}^{2}\right]$$

where σ*_ϕ_* = σ*_0_* (1 − *q*_1*ϕ*_) is the effective stress at yield for a rubber-toughened polymer containing a volume fraction *ϕ*, when the mean normal stress σ*_m_* and the void content *f* are both zero. In this equation, σ*_m_* is the yield stress of the pure matrix at σ*_m_* = *f* = 0. The factors *q*_1_ = 1.375 and *q*_2_ = 0.927 were introduced to improve the fit between Gurson’s predictions and data from numerical analysis [19]. Following dilatational yielding, the measured activation volume *V_m_* increases with the volume fraction of voids, *f*, according to the following relationship:

(5) $$V_{m}=V\left(1+2f\right)$$

This equation shows that the presence of voids significantly affects the rate of yielding as indicated by the increase in apparent activation volume.

If the dominating micromechanical deformation process is the rubber particle debonding, the stress necessary to initiate debonding, the number of debonded particles, and the size of the voids formed can be described by the following equation proposed by Pukanszky and Voros [12]:

(6) $$\sigma^{D}=-C_{1}\sigma^{T}+C_{2}{\left(\frac{W_{AB}E}{R}\right)}^{\frac{1}{2}}$$

where *σ^D^* and *σ^T^* are debonding and thermal stresses, respectively, W_AB_ is the reversible work of adhesion, and R denotes the radius of the particle. *C*_1_ and *C*_2_ are constants which depend on the geometry of the debonding process. 

## 3. Materials and Methods

### 3.1. Materials

The materials used in this work for the binary blends production were:

- Poly(lactic) acid (PLA), trade name Luminy LX175, purchased from Total Corbion PLA. It is a biodegradable PLA, derived from natural resources, that appears as white spherical pellets. According to the datasheet producer, this PLA contains approximately 4% of D-lactic acid units and can be used alone or blended with other polymers or additives for the production of suitable blends and composites. This PLA grade can be processed easily on conventional equipment for film extrusion thermoforming or fiber spinning (density: 1.24 g/cm^3^, melt flow index (MFI) (210 °C/2.16 kg): 6 g/10 min);
- Poly(butylene succinate-co-adipate) (PBSA), trade name BioPBS FD92PM, purchased from Mitsubishi Chemical Corporation, is a copolymer of succinic acid, adipic acid, and butandiol. It is a soft and flexible semicrystalline polyester that can be blended in extruder with other polymers but can be also processed by blown and cast film extrusion (density of 1.24 g/cm^3^, MFI (190 °C, 2.16 kg): 4 g/10 min).

### 3.2. Methods

#### 3.2.1. Blends and Samples Preparation

Two different binary blends compositions containing, respectively, 15 and 20 wt.% PBSA (see Table 1 for the blends’ names and compositions) were produced in pellets using a Comac EBC 25HT (L/D = 44) (Comac, Cerro Maggiore, Italy) twin screw extruder. The samples were named PLA (pure PLA), 85-15 (blend of PLA and PBSA 85/15 by weight) and 80-20 (blend of PLA and PBSA 80/20 by weight). Before the extrusion the materials were dried for 12 h in a DP 604–615 dryer (Piovan S.p.A., Verona, Italy). PLA granules were introduced into the main extruder feeder, while PBSA granules were fed into a specific side feeder. The temperature profiles of the extruder (11 zones) used for blends preparation were 150/175/180/180/180/185/185/185/185/185/190 °C, with the die zone at 190 °C. A screw rate of 300 rpm and a total mass flow rate of 20 kg/h were set. The strands coming out from the extruder were rapidly cooled in a water bath and then cut into pellets by an automatic knife cutter. All pellets were finally dried again at 60 °C.

After the extrusion, the extruded pellets were injection molded in a Megatech H10/18 injection molding machine (TECNICA DUEBI s.r.l., Fabriano, Italy) to obtain ISO 527-1A dog-bone specimens (width: 10 mm, thickness: 4 mm, length: 80 mm) for tensile tests. From the injection molding parameters (Table 1), it can be observed that the same temperature profile was adopted for all blends as well as the same cooling time was set. The mold temperature was also lowered progressively with the increasing amount of PBSA from 70 °C for pure PLA to 55 °C for 80-20. The PBSA addition causes a decrement in viscosity that results in a lowering of the injection pressure [28].

#### 3.2.2. FT-IR Characterization

Infrared spectra of pure PLA, PBSA, and PLA/PBSA blends were recorded in the 550–4000 cm^−1^ range using a Nicolet 380 Thermo Corporation Fourier Transform Infrared (FTIR) Spectrometer (Thermo Fisher Scientific, Waltham, MA, USA) equipped with smart Itx ATR (attenuated total reflection) accessory with a diamond plate. Two hundred and fifty-six scans at a 4 cm^−1^ resolution were collected. The analysis was performed on the material sampled on the gate region of injection molded specimens.

#### 3.2.3. Mechanical Characterization

Tensile and creep tests were carried out on ISO 527-1A dog-bone specimens using an MTS Criterion model 43 universal testing machine (MTS Systems Corporation, Eden Prairie, MN, USA) equipped with a 10 kN load cell and interfaced with a computer running MTS Elite Software. Tests were conducted, at room temperature, 3 days after the injection molding process and during this time the specimens were stored in a dry keeper (SANPLATEC Corp., Osaka, Japan) at a controlled atmosphere (room temperature and 50% humidity).

For standard uniaxial tensile tests, at least ten specimens for each blend composition were tested at a constant crosshead speed of 10 mm/min. The average values of the main mechanical properties were reported.

In order to investigate the nature of the deformation process, constant-load creep tests were carried out at room temperature at different stress levels, below the yield stress value, from 10 up to 40 MPa. The initial load for obtaining the set stress was reached by subjecting the specimen to uniaxial test at a speed of 10 MPa/min and, subsequently, maintaining the applied load for 8 h. Once the desired load was reached, the variation in deformation over time was recorded. Furthermore, to estimate the volume change that occurred during the creep test, transversal and axial specimen elongation were recorded with a video extensometer (Genie HM1024 Teledyne DALSA camera) interfaced with a computer running ProVis Software (Fundamental Video Extensometer) which, in turn, is interfaced with the MTS Elite Software. The volume strain (Δ*V/V*_0_) was calculated, assuming equal the two lateral strain components, according to the following equation [20,50,51]:

(7) $$\frac{\Delta V}{V_{0}}=\left(1+\varepsilon_{1}\right){\left(1+\varepsilon_{2}\right)}^{2}-1$$

where the volume variation is ∆*V*, the starting volume is *V*_0_, *ε*_1_ is the axial (or longitudinal) strain, and *ε*_2_ is the lateral strain.

In order to verify the reproducibility of the results obtained, at least 3 tests for stress level were carried out for each formulation.

#### 3.2.4. Optical Analysis

The fracture surface of the specimens subjected to creep offers reliable information about the micromechanical deformations that occurred during the creep tests. Consequently, some specimens, appropriately selected at certain stress levels, were cold fractured along the tensile direction. The fracture surfaces, coated prior with a thin layer of platinum to avoid charge build up, were investigated by an FEI Quanta 450 FEG (Thermo Fisher Scientific, Waltham, MA, USA) scanning electron microscope (SEM).

## 4. Results

**Figure 1:**
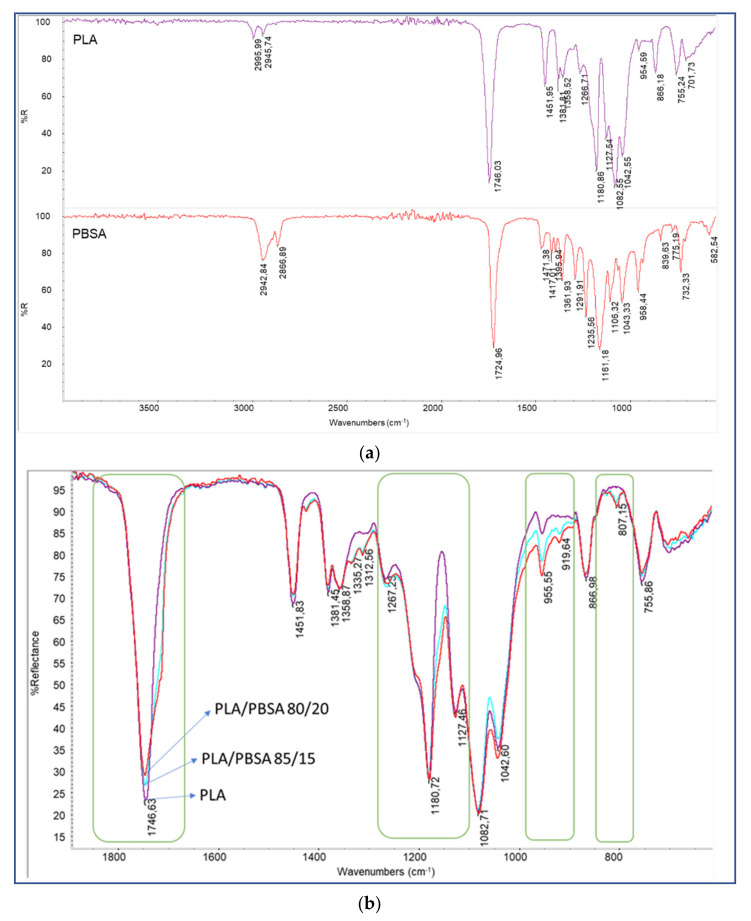
(**a**) ATR spectra of pure PLA and PBSA; (**b**) ATR spectra of PLA-PBSA blends.

After the preparation of the specimens for investigation regarding creep behavior, the blends and the pure polymers were characterized by infrared ATR spectroscopy (Figure 1).

The pure PLA showed an infrared spectrum with main bands at 2996 and 2946 cm^−1^ (stretching C–H), 1746 cm^−1^ (stretching C=O), 1180 cm^−1^ (stretching C–O–C), and 1082 cm^−1^ (stretching O–C–C–) [52,53]. PBSA, being, like PLA, an aliphatic polyester, showed similar bands, but shifted at lower wavenumbers because of the higher macromolecular flexibility of PBSA [54], being 2943, 2867, 1725, 1161, and 1043 cm^−1^, respectively. The blends PLA/PBSA 85/15 and 80/20 blends showed a spectrum much more similar to pure PLA, but some differences could be noticed. In the blend, the main stretching C=O band at 1746 cm^−1^ showed a shoulder at lower wavenumbers attributable to the presence of the C=O stretching band of PBSA. Moreover, a similar trend could be observed for the band at 1180 cm^−1^, showing a shoulder at lower wavenumbers due to the overlapping of the 1161 cm^−1^ band of PBSA. The 955 cm^−1^ was more intense in blends because PBSA showed this band, attributable to the –C–OH bending in the carboxylic acid groups of PBS [55] or vinyl esters [56] more intense than PLA. Moreover, the band at 806 cm^−1^ (present in the spectrum of pure PBSA at 840 cm^−1^) was attributable to the presence of vinyl ester moieties [57]. In general, the ATR evidence resulted in good agreement with the selected compositions of PLA/PBSA blends.

In particular, the infrared characterization did not show any evident chemical change in the two polymers; this is can be ascribed to the very short processing time. In fact, significant transesterification occurs in the presence of proper catalysts and for processing times longer than 10 min [58]. Moreover, in the literature, it has been observed by Ding et al. [59] that the formation of copolymers between PLA and PBSA during extrusion and injection molding can be considered negligible. In addition, any eventual degradation during processing can be excluded due to the processing temperature adopted that did not overcome 190 °C. For the pure polymers and their blends, in fact, it was observed that the onset temperature for pure PLA was 274 °C, and the temperature at which the maximum degradation rate was reached (inflection point) was 354.5 °C; while for PBSA, these two temperatures were shifted at 301 and 401 °C, respectively, indicating a higher thermal stability for this polymer [60,61,62].

From the tensile tests (reported in Table 2), it can be observed that the addition of PBSA makes the material more ductile. Pure PLA is a fragile material characterized by an elevated modulus of 3.58 GPa, a high tensile strength of 62 MPa, and a low elongation at break (3.6%). The mechanical results are in accordance to what can be observed in the literature [28,62,63]; the introduction of the rubbery PBSA phase at 15 and 20 wt.% led to a decrement in the Young’s modulus and tensile strength counterbalanced by an increment of the elongation at break. These trends are more marked where the PBSA content was higher (20 wt.%). With the addition of PBSA, the material yielded with the appearance of the neck that propagated along the gauge length.

**Figure 2:**
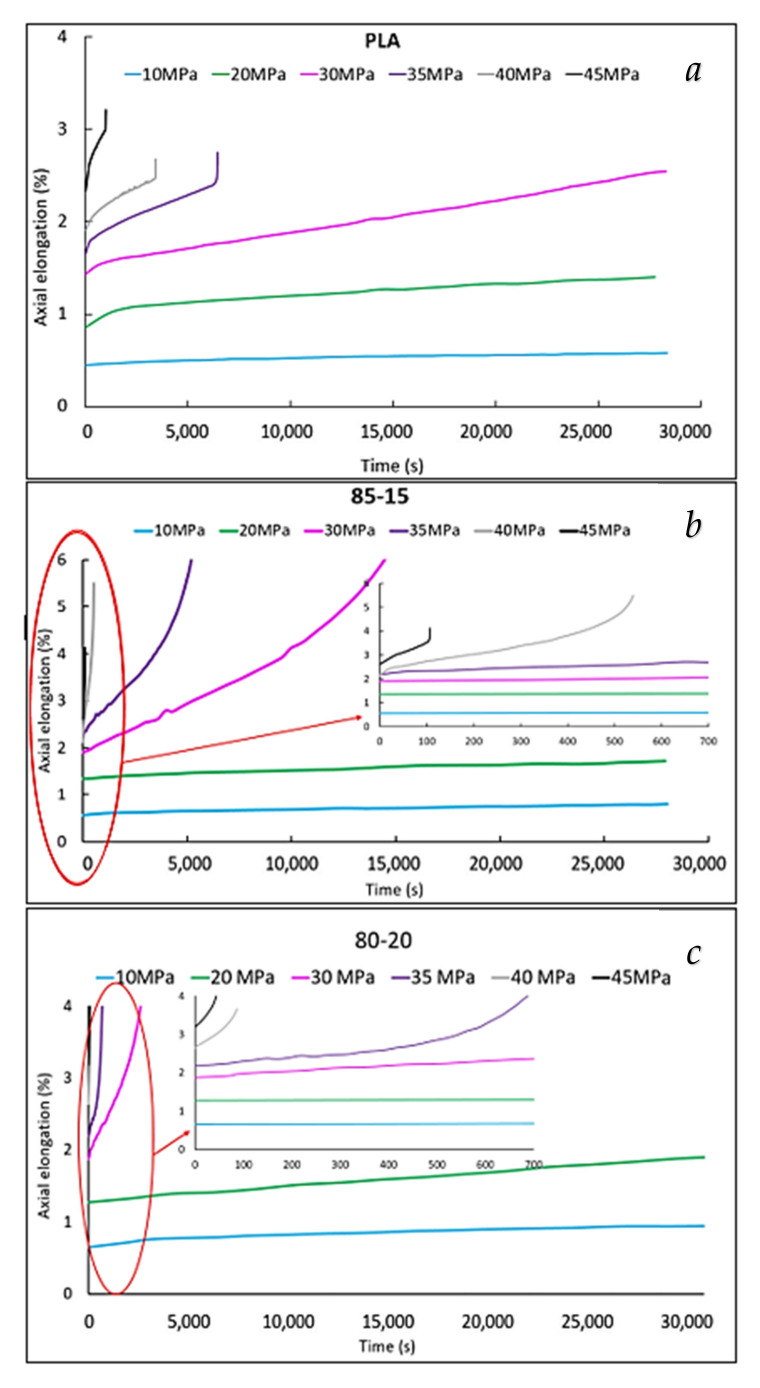
Axial elongation vs. time (creep curves) curves of pure PLA (**a**), 85-15 (**b**), and 80-20 (**c**) for different applied stresses from 10 to 45 MPa.

Standard creep curves at different stress levels are reported in Figure 2.

At stresses of 10 and 20 MPa, all the specimens did not break during the test time interval (set at 8 h). However, when increasing the PBSA content, the creep resistance decreased. In fact, pure PLA did not break even at 30 MPa, while the 85-15 and 80-20 blends began to break (8 h before) already at 30 MPa. Moreover, at the same stress level applied, the time at which breakage occurred decreased with the increase in the rubber content.

**Figure 3:**
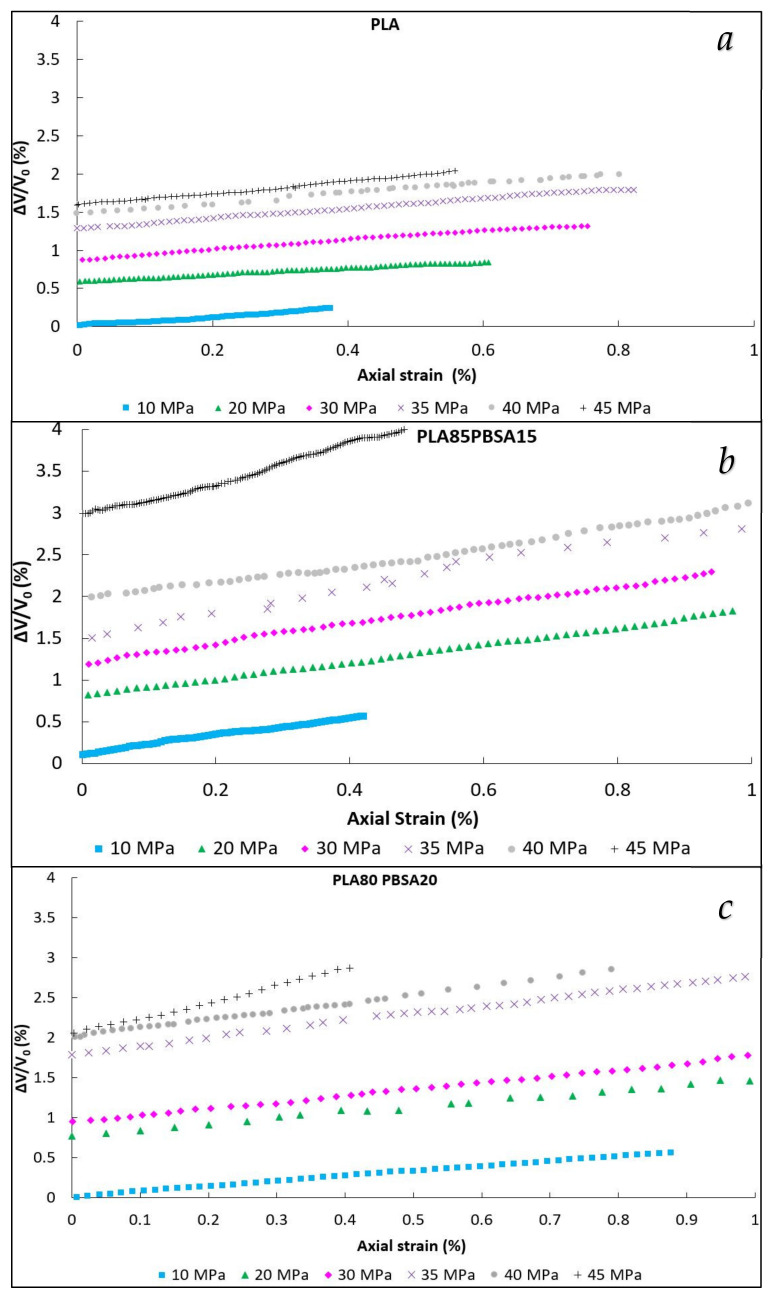
Volume variation vs. axial strain curves (dilatometric curves) of pure PLA (**a**), 85-15 (**b**), and 80-20 (**c**).

The results of the volume variations recorded after that the specimens reached the set stress levels (reported in Figure 3) showed an almost linear variation in the volume change with time. The volume variation at time zero (ΔV/V_0,i_) corresponded to the intercept of the *y*-axis in the ΔV/V_0_ vs. the axial strain curve. ΔV/V_0,i_ can be linked to the volume variation caused by the instantaneous elastic deformation of the specimen at the selected stress level applied. Increasing the stress level, the instantaneous volume change increased in accordance with the increment in the instantaneous elongation.

The volume change did not increase proportionally with the rubber content but, on the contrary, a greater volume variation was encountered for the 85-15 blend. This trend can be observed both from Figure 3 but also from the volume variation at time zero reported in Table 3 in which the slopes of the straight lines passing through the experimental data are also reported. For pure PLA, the slope remained almost constant with a slight increment with the stress level. For the 85-15 and 80-20 blends, at higher stresses, the volume strain increased more rapidly with higher slopes values. The introduction of PBSA made a substantial contribution to the dilatational processes that were more marked for the 85-15 composition.

In order to better understand the volume variation curves, it is necessary to investigate deeper the micro-mechanics of the deformation process and how the PBSA addition influences the micromechanical behavior of the binary blend.

**Figure 4:**
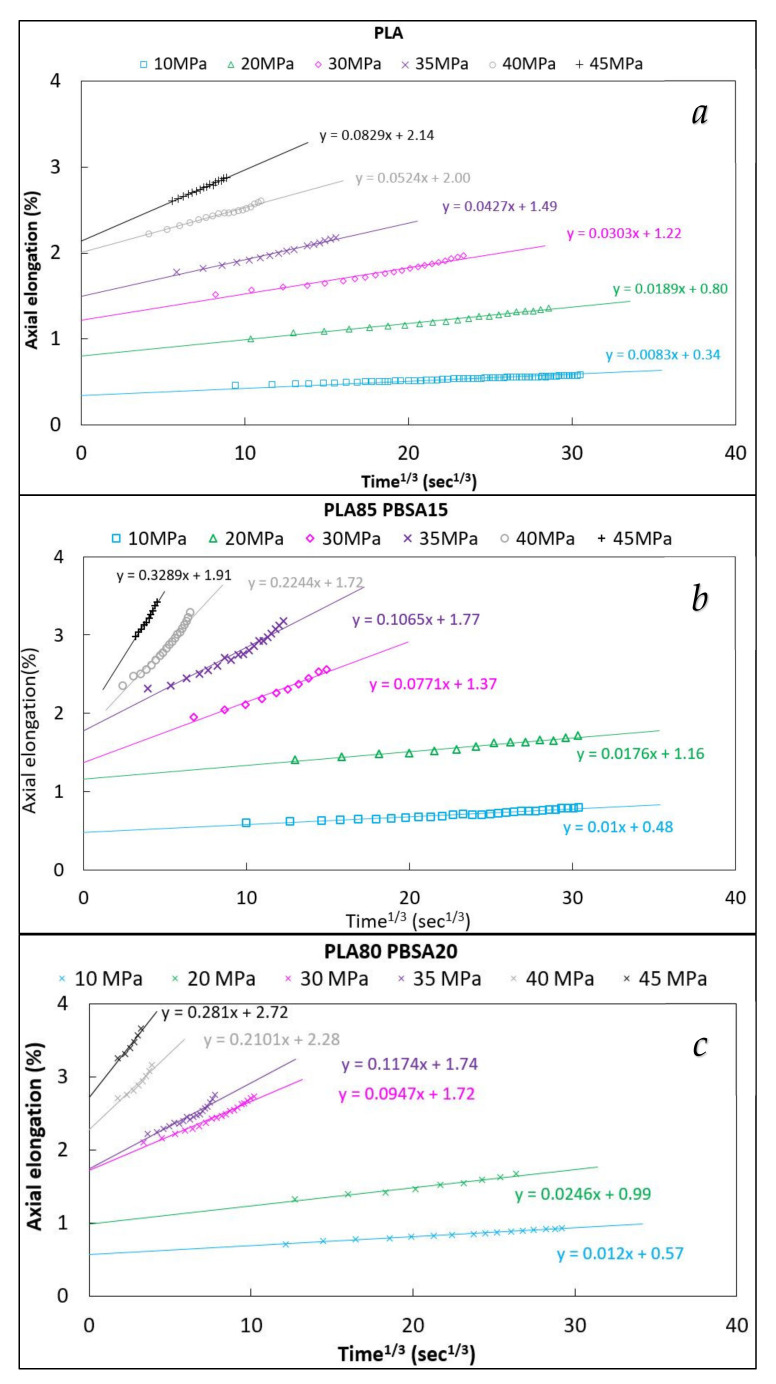
Andrade equation fittings (axial elongation vs. cubic root time) of pure PLA (**a**), 85-15 (**b**), and 80-20 (**c**).

For all compositions, it can be observed in Figure 4 that the linear region of the creep curves can be estimated, with a good fitting, by the Andrade equation (Equation (1)); consequently, for all the stress levels applied, the Andrade b parameter for pure PLA and its binary blends can be obtained, making the linear regression of the ε(t) against t^1/3^ plots.

Applying the Eyring relationship (Equation (2)) between the applied stress, *σ*, and the *b* parameter, interesting results can be obtained plotting (Eyring plot) *log b* against the applied stress, *σ*. It can be observed from Figure 3, that the Eyring plot for pure PLA gives a straight line in accordance with the Eyring model for stress activated flow. This result is also in accordance to what was observed for pure PA66 in a rubber-toughened polyamide 6,6 system [48]. For pure matrix, due to the absence of rubber PBSA particles, the stress concentration factor, γ, can be assumed equal to 1, and the activation volume, *V,* for pure PLA can be easily obtained from the Eyring plot slope. An apparent activation volume of 0.25 nm^3^ was obtained.

Under 20 MPa and 30 MPa for the 85-15 and 80-20 blends, respectively, the Eyring plots behaved in a similar manner to pure PLA (following Equation (2)) but with a slightly increased slope. An explanation for this behavior was found to be in accordance with the literature [48] and was ascribed to the same deformation mechanisms that operate in both pure PLA and PLA–PBSA blends having the same value of activation volume, *V*. The higher slope was ascribed to an increased value of the stress concentration factor, γ. On this basis, using the value of the activation volume found for pure PLA, the stress concentration factor for the PLA–PBSA binary blends can be easily obtained from the Eyring slope until 20 MPa and 30 MPa. For the two blends, 85-15 and 80-20, the stress concentration factor values obtained were very close to each other (γ = 1.15 for 85-15 and γ = 1.16 for 80-20); consequently, the γ seemed not to be affected directly by the difference in volume variation observed for the 85-15 and 80-20 blends.

**Figure 5:**
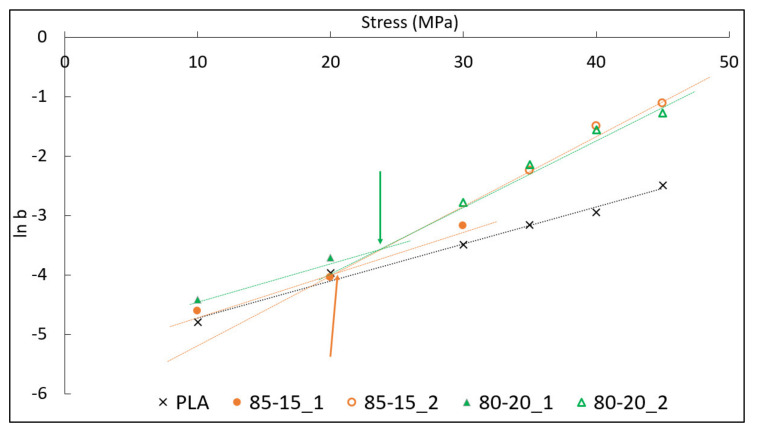
Andrade B parameter in which is highlighted the different slope changes between pure PLA and its blends (85-15 and 80-20).

However, a very interesting difference emerged from the Eyring plot (Figure 5) in which the slope changed for the binary blends occurring at two different stress levels. A small increase with no slope change could be detected over the lower range of applied stress (up to approximately 18 MPa), where, according to the literature [48], shear yielding is the predominant deformation mechanism. The stress level for which the change in slope took place can be easily identified using a simple geometric construction (the intersection point is highlighted by the green and orange arrows in Figure 5). The slope change occurred earlier (close to 20 MPa) for the 85-15 blend, while it occurred later (at approximately 25 MPa) for the 80-20 blend. The different intersection points, registered for the two binary blends investigated, were strictly connected to the volume variation differences encountered for the two types of blends. The intersection point allowed for the identification of the stress level for which the micromechanical process of debonding and/or cavitation starts and contributes to deformation. Based on the results observable in Figure 5, the debonding and/or cavitation mechanism was apparently activated earlier (at a lower stress level) for the 85-15 blend.

In fact, it must be taken into account that the creep test was carried out at a constant rate of 10 MPa/min to achieve the set stress levels. Consequently, a slightly different strain rate (of approximately 1mm/min) caused by the higher deformability of the blend that increased with PBSA content was registered. Nevertheless, the application of this method was extremely useful, because it allowed to highlight the connection between the micromechanical processes and their volume variation caused by the introduction of a rubber dispersed phase into a polymeric matrix. The voids generated by the cavitation/debonding mechanism caused the increase in the apparent activation volume, since voids allow plastic flow of the matrix around it more easily than an intact rubber particle. In fact, the bulk modulus of a rubber particle is very high, while for a void it is zero. Thus, the increased local strain rate of the polymer matrix was due to the fact that the material was no longer a “real” continuum on a microscopic scale. The deformation involves a macroscopic volume increase due to the growth of the voids generated around or inside the rubber particles, which is favored by the high level of triaxiality, leading to a nonlinear yield curve. In contrast to the case of a “continuum” polymer, where high levels of triaxiality favor crazing and cleavage mechanisms over shear yielding, for a “porous” polymer, a high triaxiality considerably accelerates plastic flow. 

**Figure 6:**
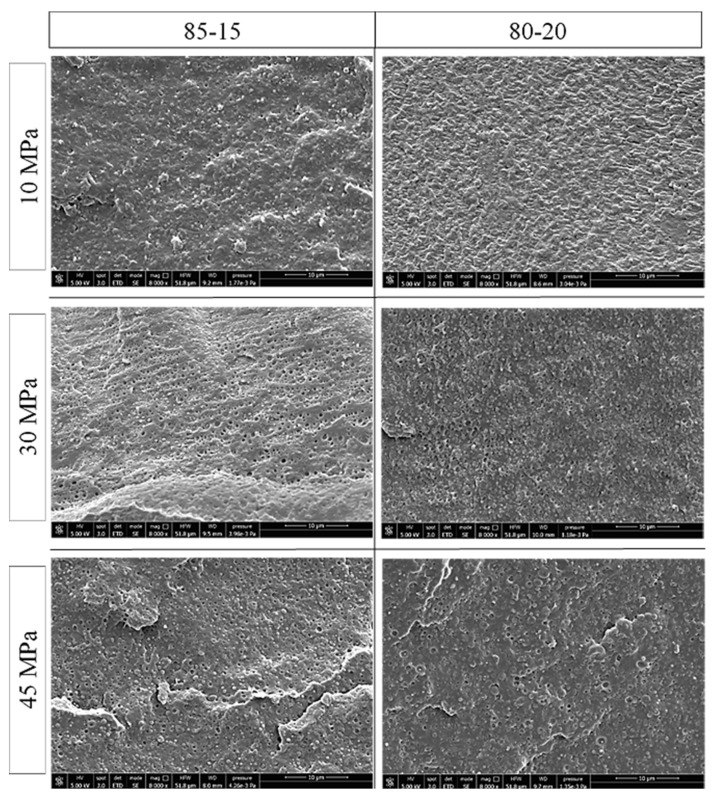
SEM micrographs made at the surface of tensile specimen cryo-fractured along the draw direction at different creep stress levels.

In order to confirm the results obtained and to understand if PBSA particles undergo debonding or cavitation, SEM micrographs (at 8000 X, Figure 6) at the surface of the tensile specimen, cryo-fractured along the draw direction and at different stress levels, were carried out.

On the basis of the results obtained in Figure 5, the most significant stress levels were analyzed. In particular, three stress levels were chosen: (I) 10 MPa, which was the lowest value for which both the binary blends underwent the same deformation mechanism and no deviation in the Eyring’s slopes occurred; (II) 30 MPa in which the slope variation occurred for both 85-15 and 80-20; (III) 45 MPa, which was the highest stress level tested and for which the micromechanical deformation process should be more marked.

From the SEM images shown in Figure 6, it emerges that the PBSA particle size increases with the PBSA content, in agreement to what is also observed in the literature [28]. At 10 MPa, for both blends, neither cavitation nor debonding of the PBSA particles appeared. At this stress level, only shear yielding took place. The fracture surfaces were characterized by well dispersed PBSA spherical particles that were well attached into the PLA matrix. The matrix showed signs of deformation caused by the creep test with greater matrix deformation occurring for the 80-20 composition in which the rubber amount was higher.

At 30 MPa, it was evident that the micromechanical deformation that occurred was caused by the debonding of the PBSA particles. Clear visible voids around the PBSA particles could be distinguished. At 30 MPa, therefore, despite 80-20 having a higher PBSA content, the 85-15 blend had the greatest number of particles that underwent debonding. This result is in agreement to what emerged from the Eyring’s plot; in fact, for the 85-15 composition debonding started at a lower stress level, generating at 30 MPa a greater number of debonded particles. By increasing the stress level of the creep test (up to 45 MPa), debonding continued to be the main micromechanical deformation mechanism for both the binary blends. Increasing the stress level, the quantity of particles that underwent debonding also increased.

## 5. Conclusions

Over the last years, there has been a growing interest toward biobased and biodegradable polymers that show a more environmentally friendly end of life and, at the same time, are able to improve carbon neutrality when compared to their fossil-based counterparts. In order to improve the physical and mechanical properties of these biopolymers, a blending technique is fundamental. For this purpose, a better knowledge of the micromechanical deformation processes of these new biopolymeric blends is fundamental in order to better exploit their peculiar properties, favoring the replacement of their fossil-based counterparts in several application sectors.

Despite different studies that can be found in the literature on biopolymeric blends, an in-depth investigation of micromechanical mechanisms has not yet been considered. In this study, poly(lactic acid) (PLA) and poly(butylene succinate-adipate) (PBSA) blends were investigated with a PBSA content of 15 and 20 wt.%, respectively (named 85-15 and 80-20). Measurements of the volume strain, using an optical extensometer, were carried out with a universal testing machine in creep configuration trying to determine, accompanied by SEM images, the micromechanical deformation processes involved in the biopolymeric blend systems. Analytical models were also applied to correlate the creep to the dilatation variation; in particular, the Andrade equation was applied and the *b* parameter for the polymeric systems was calculated. Plotting the *log b* against the applied stress, using an Eyring plot, a significant change in the curves was found. The binary blends, in fact, showed a sharp increase in *dlogb/dσ* where significant dilatation began. The point at which the slope change occurred reasonably coincided with the onset of the cavitation/debonding mechanism. For both binary blends, the SEM analysis evidenced that the starting micromechanical deformation mechanism responsible for the volume increment was due to the PBSA debonding. 

Another interesting result obtained by applying the Eyring relationship was the calculation (knowing the data of the pure PLA matrix) of the stress concentration factor; it emerged that the second PBSA phase acts as a stress concentrator, probably due to the presence of weak interfaces between PLA and PBSA that are also responsible for the debonding mechanism.

## Figures and Tables

**Table 1:** Injection molding conditions.

Parameters	PLA	85-15	80-20
Temperature profile (°C)	180/185/190	180/185/190	180/185/190
Mold temperature (°C)	70	60	55
Injection holding time (s)	5	5	5
Cooling time (s)	15	15	15
Injection pressure (bar)	90	80	80

**Table 2:** Tensile tests results.

Blend Name	Young’s Modulus (GPa)	Stress at Break (%)	Elongation at Break (%)	Yield Stress (MPa)	Elongation at Yielding (%)
PLA	3.58 ± 0.04	61.58 ± 0.87	3.57 ± 0.23	-	-
85-15	2.89 ± 0.02	20.56 ± 1.35	62.71 ± 16.63	55.05 ± 0.66	4.18 ± 0.04
80-20	2.75 ± 0.07	19.51 ± 0.49	71.67 ± 11.92	51.07 ± 0.86	4.12 ± 0.11
PBSA	0.25 ± 0.04	23.4 ± 0.55	898.92 ± 21.03	16.6 ± 0.22	29.02 ± 0.23

**Table 3:** Volume variation at time zero and the slopes of the ΔV/V0 vs. the axial strain curve at different stress levels.

Blend Name	ΔV/V_0,i_ (%)	Slope of the ΔV/V_0_ vs. Axial Strain Curve	Stress Level (MPa)
PLA	0	0.5	10
	0.59	0.55	20
	0.88	0.61	30
	1.29	0.65	35
	1.43	0.66	40
	1.59	0.78	45
85-15	0.12	1.03	10
	0.8	1.05	20
	1.21	1.14	30
	1.6	1.22	35
	1.94	1.24	40
	2.93	2.22	45
80-20	0.02	0.63	10
	0.76	0.72	20
	0.94	0.83	30
	1.79	1	35
	2.01	1.04	40
	2.03	2.04	45

## Data Availability

The data presented in this study are available on request from the corresponding author.
